# Hybrid multi-resolution network for DAS data denoising

**DOI:** 10.1371/journal.pone.0325299

**Published:** 2025-06-10

**Authors:** Li Ding, Haoran Sun, Haoliang Chen, Xinyu Hu

**Affiliations:** 1 School of Software Engineering, Jilin Technology College of Electronic Information, Jilin, China; 2 China of Limited Company of Jilin Province Power Communication Company, Changchun, China; 3 Northeast Electric Power Design Institute CO., LTD. of China Power Engineering Consulting Group, Changchun, China; Islamia University of Bahawalpur: The Islamia University of Bahawalpur Pakistan, PAKISTAN

## Abstract

The rapid advancement of Distributed Acoustic Sensing (DAS) technology has opened up extensive prospects within the field of seismic exploration. However, unforeseeable noise present in actual DAS seismic records has led to the submergence of valuable information beneath intense noise, significantly disrupting reflective signals and diminishing the signal-to-noise ratio (SNR) of seismic data. Consequently, subsequent processing, such as migration and imaging, and interpretation tasks are hindered. In pursuit of an effective denoising approach for DAS data, this study proposes a Hybrid Multi-Resolution Network (HMR-Net), which concentrates on extracting coarse or intricate features from multi-resolution feature maps, thus delving into profound seismic characteristics across diverse scales and resolutions. The integration of error-resilient up-sampling and down-sampling processes serves to optimize the feature extraction ability and mitigate losses arising from sampling procedures. Furthermore, a highly authentic dataset was compiled by utilizing real DAS noise data along with synthetic records obtained through forward simulations. Through validation against both synthesized records and actual seismic records, the effectiveness of the proposed approach in substantially suppressing noise and enhancing the SNR has been demonstrated.

## 1. Introduction

Distributed Acoustic Sensing (DAS) technology is an innovative acquisition method that captures subtle strains induced by seismic waves through recording the phase of scattered light [1]. This technique offers advantages such as low acquisition costs and small sampling intervals, rendering it highly promising within the field of seismic exploration [2]. Nonetheless, during actual record processes, the sensitivity of scattered light phase in optical fibers makes the acquisition vulnerable to background noise [3]. The background noise severely degrades the scattered signals, causing weak signal components heavily influenced by intense noise. Consequently, the quality and integrity of the acquired seismic records is substantially compromised.

Compared to conventional seismic data, the novel acquisition technique brings new types of interference and present complicated features, such as optical noise, fading noise, horizontal noise, and coupling noise [4,5]. Under the influence of these intense noise, the signal-to-noise ratio (SNR) of DAS records experiences a notable reduction. Enhancing the SNR of DAS records has become a pivotal topic of seismic signal processing. Notably, it also profoundly affects subsequent data analysis and interpretation processing [6]. Therefore, a detailed investigation on noise reduction and signal recovery techniques for DAS records has great practical significance, considering the compliexity of noise features and their detrimental effects on data integrity [7].

Over the past few decades, numerous conventional signal processing methods have been employed to enhance the SNR of seismic records. These methods include spatial domain filtering techniques such as median filtering [8], weighted median filtering [9], mean filtering [10], Wiener filtering [11], and compressed sensing [12]. Typically, these conventional methods utilize convolution and correlation operations to perform blurring or sharpening in the spatial domain of the image.While they exhibit a particular efficacy against specific types of noise, seismic records are often characterized by intense and intricate noise amplitudes, rendering spatial domain filtering less effective. Consequently, transform domain filtering methods, capable of accommodating the time-frequency characteristics of noise, have been integrated into seismic denoising. Techniques such as Short-Time Fourier Transform [13], Wavelet Transform [14], and bandpass filtering [15] have been applied in this aspect. Bandpass filtering exhibit effective noise removal capabilities for high and low-frequency noise; however, their performance in filtering noise of the same frequency is limited. Short-Time Fourier Transform employs a fixed time-frequency window, thereby compromising its ability to capture fine details in both domains simultaneously. Wavelet Transform offers rapid computation and simple application, yet it places significant demands on threshold selection. In essence, transform domain filters aim to decompose signals within a transform domain equivalently. This involves distinguishing between noise and valuable signals within that domain using thresholds, and then recontructs the signal. However, the extensive overlap between noise and valuable signals in seismic records hinders the effectiveness of this approach. Furthermore, methods based on sparse representation, such as the basis pursuit algorithm (BP) [16], K-singular value decomposition [17], and fixed dictionaries [18], leverage the characteristic that proper signals can often be sparsely represented, whereas random noise typically lacks sparse representation. These techniques are gradually finding applications in seismic denoising; however, the outcomes were degraded in some conditions.

In recent years, with the advancement of deep learning, convolutional neural networks (CNNs) have been introduced into the domain of seismic noise reduction [19–21]. Generally, these approaches aim to establish high-dimensional, nonlinear mappings between noisy records and clean signals through the training process [22,23]. This enables the extraction of useful signals from noisy records, thus achieving the objective of noise reduction. Representative examples include DnCNN [24] and U-Net architectures [25]. Although these methods exhibit significant improvements over conventional approaches in effectiveness, preliminary experiments indicate that most of these techniques still fall short of meeting the requirements imposed by DAS records. The limitations of these frameworks primarily stem from two key factors: Firstly, conventional multi-scale network architectures exhibit diminished performance in complex feature extraction due to their simplistic feature interaction strategies. For another, the training dataset cannot provide enough informative and significant features in real seismic data, thereby bringing negative impacts on the denoising performance. Therefore, the further attempts of network architecture with enhanced feature extraction capabilities remains pivotal for DAS record denoising.

In this paper, we propose a Hybrid Multi-Resolution Network (HMR-Net), which augments the U-Net architecture with a Multi-Resolution (MR) feature extraction module. To address the limitations above, the contributions of HMR-Net are concentrated on the following aspects: Taking the network design as an instance. Using both up-sampling and down-sampling, the network continuously extracts diverse multi-scale features from the fusion of high and low-resolution feature maps. The integration of hybrid resolutions renders this network more targeted, thereby ensuring its denoising capability. In the construction of training data, a dataset well-aligned with seismic data characteristics was generated. This dataset incorporates synthetic reflection signals combined with real background noise, thus ensuring the network’s generalization capability. To evaluate the network’s performance, we applied it to process both synthetic and field seismic data. Moreover, we conducted comprehensive comparisons between the results achieved by this network, traditional methods, and recent CNN-based approaches. The outcomes reveal that HMR-Net exhibits certain advantages in mitigating DAS background noise and preserving small signal amplitudes.

The main contents of the study can be concluded as follows: In section 2, we introduce the network architecture and denoising principle of HMR-Net. In section 3, the training details and processing results on synthetic DAS data is given. In section 4, the denoising performance on field DAS data is analyzed. Morevoer, we also compare HMR-Net with DeepSeg to further evaluate the denoising capability in section 5. Finally, section 6 summarizes the findings and contributions of this study.

## 2. Method

In DAS records, valuable information is often obscured by strong noise. Single-scale extraction frameworks, such as DnCNN [24], may yield unsatisfactory results. Therefore, how to effectively utilize the potential features existed in multi-scale information has become a feasible approach. In this study, HMR-Net is designed and applied to attenuate the intense background noise in DAS data. The architecture of network components and denoising princile are described as follow.

### 2.1. Network architecture

**Fig 1:**
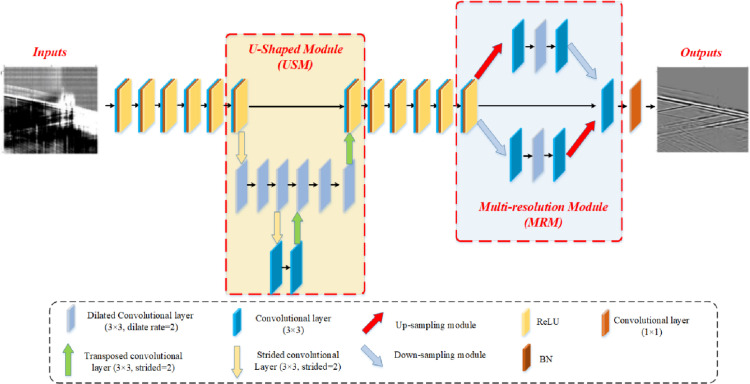
Structure of HMR-Net.

**Fig 2:**
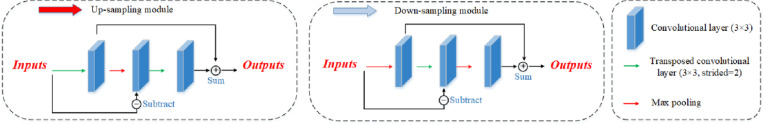
Structure of Up-sampling and Down-sampling module. (a) Up-sampling module. (b) Down-sampling module.


As shown in Fig 1, HMR-Net is built upon a feedforward neural architecture, utilizing diverse convolutional operations to ensure the extraction of multi-scale features. Generally, the input data is firstly processed by five convolutional layers (kernal size is 3 × 3) combined with batch normalization (BN) layers and ReLU function, aiming to preliminarily extract the potential features. On this basis, an U-shaped module (USM) is utilized capture the multi-scale information. Notably, the up- and down-sampling processes are accomplished by transposed convolutional layers and strided convolutional layers, respectively. Meanwhile, we use 3 × 3 dilated convolutional layers (dilated rate equals 2) to accelerate the feature extraction. Here, we aim to use global information existed in low-resolution data to help the feature extraction in high-resolution data. In other words, the incorporation of multiple rounds of up-sampling and down-sampling preserves multi-resolution attributes, enabling the targeted extraction of both coarse and detailed features inherent in DAS data. After USM, we use another four convolutional layers combined with BN and ReLU function to refine the captured multi-scale features. Another issue need pay further attention is the large amplitude scope of field DAS data, and the recovery of weak signals is challenging. To solve this problem, multi-resolution extraction module (MREM) is designed. Here, we use up-sampling and down-sampling module, whose architectures are shown in Fig 2, to obtain high-resolution and low-resolution features. Specifically, we use two 3 × 3 convolutional layers and a dilated convolutional layer with dilated rate of 2 to refine the global and detailed features in high- and low-resolution feature maps. Then, the captured features are concatenated with the intial inputs, aiming to improve the denoising capability when confronting weak signal buried in intense background noise. Furthermore, the purpose of designing up-sampling and down-sampling modules is to ease the loss of detailed features. Notably, these modules not only employ convolution and deconvolution for up-sampling and down-sampling but also reintroduce the loss data obtained through subtraction. For a clear representation, the process of MREM can be denoted by the following equation:

$$\begin{array}{c} K_{0}=f^{1}(f^{3}(C(D_{o}(f^{3}(f_{d,2}^{3}(f^{3}(U_{p}(F)))));F;\\ U_{p}(f^{3}(f_{d,2}^{3}(f^{3}(D_{o}(F)))))))) \end{array}$$ (1)

where $f^{1}$ and $f^{3}$ represent the operation of a 1 × 1 and 3 × 3 convolutional layers, respectively. *F* stand for the input features, while C represents the concatenation operation. Moreover, *Up* and *Do* denotes the up-sampling and down-sampling operations. Furthermore, $f_{d,2}^{3}$ denotes a dilated convolutional layer with a kernel size of 3 × 3 and a dilation rate of 2. At last, a 1 × 1 convolutional layer is used to prepare to output the final denoising results. Besides, we use ADAM algorithm to optimize the learnable parameters, and the specific hyper-parameter setting for HMR-Net is shown in Table 1.

**Table 1:** Network architecture parameters.

Hyper-parameter	Specification
Optimizer	ADAM
Patch size	64 × 64
Batch size	32
Epoch	60
Learning rate range	[5,6,10]
Input channels	1
Layers	28
Convolution kernel size	3 × 3 × 64

### 2.2. Denoising theory

In the denoising tasks, a substantial portion of noise is superimposed on useful signals in an additive manner. Therefore, we can assume that the noisy DAS records, denoted as *y*, can be expressed as follows:


y=s+n (2)

Here, *s* represents the clean signal, and *n* represents the intense background noise. Subsequently, a network model can establish a nonlinear mapping from seismic record *y* to estimate the effective signal *s*. This mapping relationship is depicted in Equation 3:


$$\hat{s}=F(y;\zeta )$$ (3)

where $\begin{array}{c} \hat{s} \end{array}$ denotes the estimated value of the clean signal, *F* signifies this mapping relationship, while ζ={w,k} denotes the parameters within the neural network—weight parameters k and bias parameters. During the initial stages of training, as network parameters are usually randomized, estimation errors tend to be significant. As we know, mean-square-error (MSE) metric is commonly used in signal processing, which can effectively evaluate the accuracy of estimation or reconstruction results. To minimize the estimation errors, a MSE-based loss function is applied, defined as follows:


$$L(\zeta )=\frac{1}{N}\sum_{i=1}^{N}{\left\|F(y_{i};\zeta )-S_{i}\right\|}^{2}$$ (4)

where $y_{i}$ is the noisy records within the dataset, and $S_{i}$ denotes the labeled signal components. Consequently, this network is designed to learn features from the clean signals. Through iterative refinement of network parameters, it gradually approaches the goal of estimating values that closely approximate the clean signals.

## 3. Network training and datasets

### 3.1. Network training process

As a state-of-the-art denoising method, deep learning’s remarkable efficacy is largely dependent on the diversity and comprehensiveness of the training dataset. The training dataset for this network consists of sets of clean signals and authentic noise signals. In previous research, clean signal sets were often simulated using typical seismic wavelets to represent effective signals. However, the generated clean signals were oversimplified, omitting aspects of propagation and reflection, resulting in a lack of weak reflection signals within the constructed clean signal sets. Consequently, post-training, the network model exhibited suboptimal performance in recovering weak reflection signals within DAS records. Therefore, to acquire a set of clean signals rich in reflection information, a large quantity of pristine synthetic records was generated using forward modeling methods. The training process encompassed a total of 60 epochs.

### 3.2. Construction of training datasets

**Fig 3:**
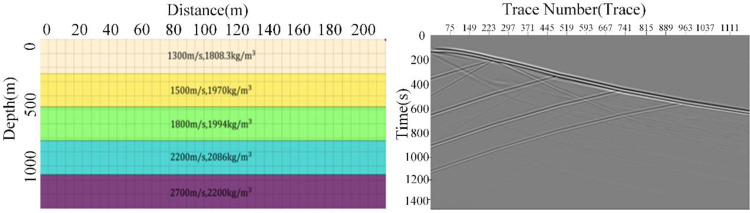
Forward models and generated synthetic record. (a) The geological models (b) The synthetic models.

**Fig 4:**
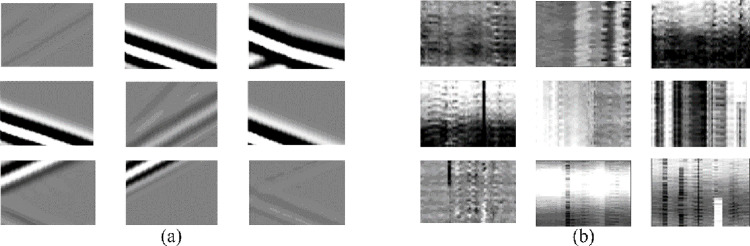
Samples of training patches. (a) Signal patches (b) Noise patches.

**Fig 5:**
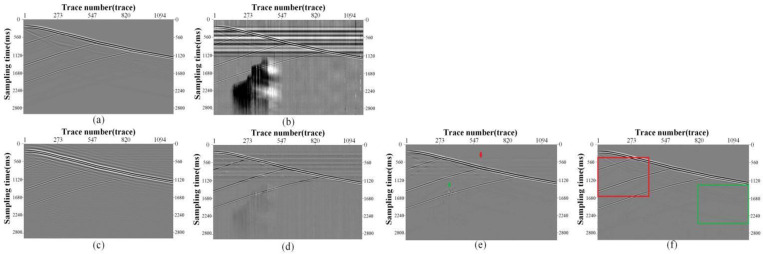
Synthetic record and denoising results. (a) Clean Signal, (b) Noisy Signal, (c) Bandpass filtering denoising result. (d) DnCNN denoising result, (e) U-Net denoising result, (f) HMR-Net denoising result.

During the construction process of the clean signal set, we referred to early exploration records from similar regions and incorporated geological conditions during exploration. By adjusting physical parameters such as wave velocity and layer thickness, we created 150 forward simulation models. The diversity of these models ensured the comprehensive representation of subsurface events within the clean signal set and bolstered the generalization capability of the training model. Subsequently, we employed seismic wavelets of various dominant frequencies to activate these forward simulation models. By utilizing the wave equation and finite difference method, we simulated and recorded wavefield data, resulting in a comprehensive set of high-quality DAS records. Fig 5 illustrates the forward modeling process. In Fig 3(a), the constructed forward stratigraphic models exhibit distinct wave velocities: 1300 m/s, 1500 m/s, 1800 m/s, 2200 m/s, and 2700 m/s, all with a consistent layer depth of 240 m. The red triangle in the upper right corner represents the position of the artificial source, while the black vertical line on the left signifies the DAS downhole receiver line. Fig 3(b) portrays the recorded wavefield information following seismic wavelet excitation. Subsequently, these records were partitioned into small signal patches of 64 × 64 dimensions, from which a random selection of 20,000 patches were chosen to form the signal set. From this set, 9 small signal patches were randomly selected, as depicted in Fig 4(a).

The noise dataset is extracted from actual DAS records with a size of 5010 × 15000, obtained from Southwest China. Then, the noise records are split into 32 × 32 segments, and 10000 patches are selected out. Here, horizontal noise is difficult to attenuate owing to its large amplitude. Aiming to improve the denoising capability, we enlarge the ratio of horizontal noise to 1/10 of the total patches. For clarity, Fig 4(b) lists 9 samples of typical noise patches. Moreover, we use a variable weights to control the amplitude of the noise patches, and then randomly add them with signal patches to generate noisy patches with different SNRs. In this study, the signal patches is used as the labels, while the noisy patches are fed into the networks. By optimizing the network parameters, we can establish a mapping from noisy data to clean signals, which is considered as trained models and further used to denoise other DAS data.

## 4. Processing and analysis of synthetic data

### 4.1. Synthetic records processing results

To evaluate the performance of our network, a synthetic clean record was generated using the aforementioned forward modeling approach, as depicted in Fig 5(a). This forward simulation model comprised four layers with wave velocities of 1500 m/s, 1800 m/s, 2200 m/s, and 2700 m/s, respectively. The layer depths were all set to 300 m. A 40 Hz Ricker wavelet was employed to excite the model. To evaluate the denoising performance, real background noise data is added to clean record to form a noisy DAS record, as shown in Fig 5(b). Notably, the upgoing events are heavily influenced by the intense horizontal noise, and the weak events in the bottom parts even submerged in the time-varying optical influence. Here, we use HMR-Net to process the noisy data. Meanwhile, some competing methods are also selected, including band-pass filtering and other CNN-frameworks. Specifically, the pass band of bandpass filtering is set to [8, 50]Hz. The competing frameworks mainly include DnCNN and U-Net, and they are trained with the same dataset and similar hyper-parameter setting as HMR-Net. Fig 5(c)–5(f) gives the denoising results of these aforementioned methods. Through observation, it is noted that the bandpass filter somewhat restored the useful signal. However, the continuity of the recovered reflection events was poor, with some distortion. Moreover, there remained noticeable residual noise, sharing the same frequency band with effective signals. The denoising effect of DnCNN exceeded that of the bandpass filter, but the resulting image still contained significant background noise, such as residual horizontal noise. In contrast, U-Net removes most of the noise, but some noise residue persisted, as the areas indicated by the red arrow and the green arrow in Fig 5(e). The results of processing the synthetic record using HMR-Net are depicted in Fig 5(f), where the background is notably clear. Moreover, the small-amplitude signals on the right side of the image, indicated by the green arrows, are well recovered.

**Fig 6:**
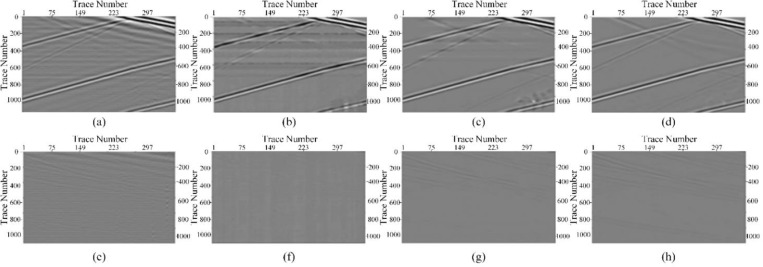
Detailed comparions for diiferent denoising methods. (a, e) Enalrged areas of bandpass filtering results. (b, f) Enalrged areas of DnCNN results,(c, g) Enalrged areas of U-Net results, (d, h) Enalrged areas of HMR-Net result.

To provide a more detailed comparison, we enlarge the areas marked by the red and green blocks, and the enlarged figures are shown in Fig 6. The bandpass filter exhibits significant aliased noise, and it is evident that the weak signals are fragmented and distorted. Both DnCNN and U-Net results exhibit considerable residual horizontal noise and background interference. Through the comparative analysis in these enlarged figures, it is evident that the processing outcome of HMR-Net has a clear background. Various types of noise have been effectively filtered out. Furthermore, as illustrated in Figs 8(e)–8(h), HMR-Net demonstrates a superior ability to recover weak signals compared to alternative methods. The weak signals recovered by HMR-Net exhibit enhanced continuity and completeness, which in turn facilitates more accurate subsequent interpretation tasks.

**Fig 7:**
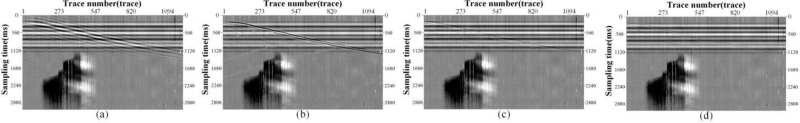
Removed noise and signal-leakage analysis. (a)-(d) Removed noise of bandpass filtering, DnCNN, U-Net and HMR-Net, respectively.

To observe the signal leakage, the removed noise is analuzed, as shown in Fig 7. It is worth noting that bandpass filtering (Fig 7(a)) and DnCNN (Fig 7(b)) significantly impact and damage the integrality of effective signals. Obvious upgoing events remain in the area dominated by the horizontal noise. In contrast, U-Net (Fig 7(c)) and HMR-Net (Fig 7(d)) do not bring conspicuous negative influences to the preservation of signal amplitude. Even so, some weak signal still can be found in the results of U-Net. Among these results, HMR-Net can provide the best performance, and almost no apparent residual signals left in the removed noise, indicating its advantages in signal preservation.

**Fig 8:**
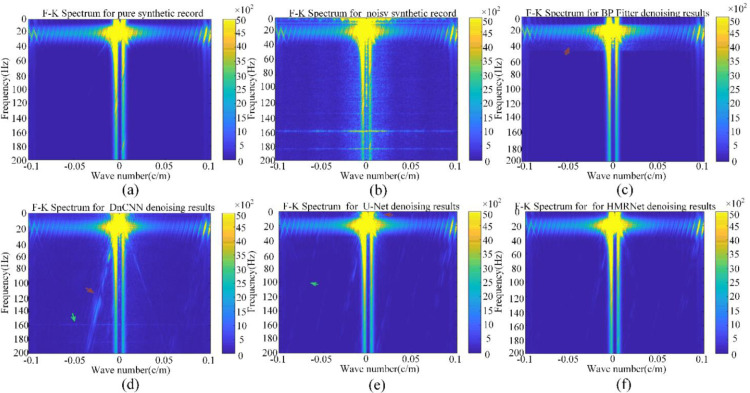
F-K spectral analysis. (a) and (b) F-K spectra for clean synthetic data and noisy record. (c)-(f) F-K spectra for the denoising results obtained by bandpass filtering, DnCNN, U-Net and HMR-Net, respectively.

To further analyze the processing accuracy, we have calculated the F-K spectra for the denoising results obtained by the aforementioned methods. Fig 8(a) and 8(b) give the F-K spectra of clean signals and noisy data. The existence of the intense noise blured the features of clean signals. On this basis, Fig 8(c)–(f) display the corresponding results obtained by bandpass filtering, DnCNN, U-Net and HMR-Net, respectively. From the result of the bandpass filter in Fig 8(c), it can be observed that low-frequency and high-frequency noise are completely suppressed. However, noise with the same frequency band as the effective signal, as indicated by the red arrow in Fig 8(c), continues to overlap with the useful signal. Moreover, DnCNN exhibits suppression of noise across all frequency bands, however, the low-frequency noise is not completely suppressed, as shown by the red and green arrows in Fig 8(d). Although U-Net can effectively suppresses the intense noise to some extent, some noise components still left, such as those marked by the green arrow in Fig 8(e). As seen in Fig 10(f), HMR-Net provide the best denoising results, and no conspicuous noise components could be observed in the F-K spectrum.

For quantitative analysis, we employ SNR [20] and Root Mean Squared Error (RMSE) [23] as the metrics. To analyze the generalization ability, synthetic noisy records with different SNR are processed, and Table 2 presents the corresponding results of denoising methods under different SNR conditions. The bandpass filter performs well in suppressing out-of-band noise; however, its effectiveness against the aliasing noise remains limited. It results in an SNR improvement of approximately 0 dB, which falls short of meeting the demands of seismic denoising task. DnCNN shows an average improvement of around 10 dB, while U-Net improves by approximately 16 dB. In contrast, HMR-Net achieves the highest increase in SNR. Another issue needs to be noticed is that HMR-Net can provide an over 20dB increment for DAS data having an SNR of -15dB. Therefore, we can get the point that HMR-Net perform better than other competing methods, particularly for the DAS data with extremely low SNR.

**Table 2:** Denoising performance of different methods.

Synthetic	BP Filter		DnCNN		U-Net		HMR-Net	
record/dB	SNR/dB	RMSE	SNR/dB	RMSE	SNR/dB	RMSE	SNR/dB	RMSE
5	3.87	0.2733	15.11	0.1169	19.21	0.1012	20.33	0.1001
0	2.34	0.3094	10.89	0.1325	17.24	0.1081	18.48	0.1056
−2	1.89	0.3861	8.22	0.1502	15.24	0.1161	16.33	0.1097
−4	1.27	0.4854	5.52	0.1755	13.08	0.1187	14.13	0.1276
−6	0.43	0.6105	2.84	0.2877	10.70	0.1331	11.93	0.1306
−8	−0.63	0.9681	0.25	0.6002	8.14	0.1507	9.75	0.1399
−10	−1.92	1.1666	−1.24	0.9833	5.62	0.1443	7.62	0.1432
−15	−5.37	1.5079	−2.22	1.2077	0.54	0.5924	3.21	0.2663

### 4.2. Ablation experiments

In this subsection, we are aiming to analyze the effects of the U-shaped module (USM) and the multi-resolution extraction module (MREM). Generally, we use the same number of stacked convolutional layers to replace these two network components, and then trained the denoising models with the the same training dataset as mentioned above. The quantitative compare results are shown in Table 3. We can find that the framework with both the U-shaped module and the Multi-Resolution Extraction module can provide the most significant SNR increment, indicating the contributions of these two network components.

**Table 3:** Comparisons of the network components used in the ablation experiment.

Module usage	Improved SNR (dB)	RMSE
USM(**×**)MREM(**×**)	12.68	0.4783
USM(**×**)MREM(**√**)	14.77	0.4126
USM(**√**)MREM(**×**)	15.31	0.4011
USM(**√**)MREM(**√**)	17.62	0.3970

## 5. Processing and analysis of field data

**Fig 9:**
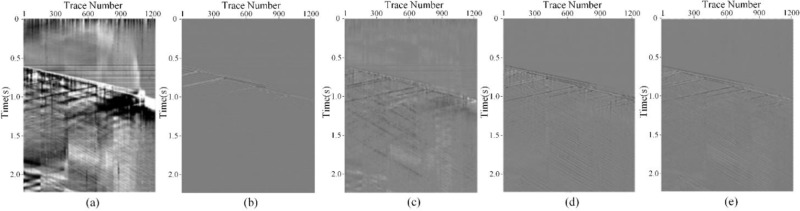
Denoising performance on field DAS data. (a) Real DAS record. (b)-(e) Denoising results obtained by bandpass filtering, DnCNN, U-Net and HMR-Net.

To evaluate the practical application of HMR-Net, this section applies HMR-Net along with other methods to process real DAS records. Fig 9(a) displays the real DAS records, which comprising 1368 traces. Moreover, each trace has a duration of 20 seconds, and the sampling frequency is 2000Hz. The weak arrivals in both the downward and upward directions are significantly obscured by noise, while the right side of the record is entirely masked by background interference. On this basis, the bandpass filter, DnCNN, U-Net, and HMR-Net are employed on this actual record. The result of the bandpass filter is shown in Fig 9(b), which corrupts the useful reflection events and introduces significant signal distortion. In contrast, CNN-based approaches recover valuable information embedded in strong noise. However, as seen in Fig 9(c), the result of DnCNN still exhibits shortcomings in noise suppression. The noise suppression effect is moderate, with a considerable amount of fading noise and horizontal noise. Moreover, the result of U-Net, depicted in Fig 9(d), also suffers from the residual noise, particularly for the incomplete reduction of horizontal noise. Notably, the outcome of HMR-Net in Fig 9(e) effectively restores valuable signals from strong noise, enhancing the visibility and continuity of seismic events. Another issue needs to be noticed is that HMR-Net can reconstruct weak reflection events, which are previously submerged in strong noise. Thus, it is evident that HMR-Net excels in background noise suppression and useful signal recovery compared to the other competing methods. This highlights its capacity to meet the processing requirements of DAS records.

**Fig 10:**
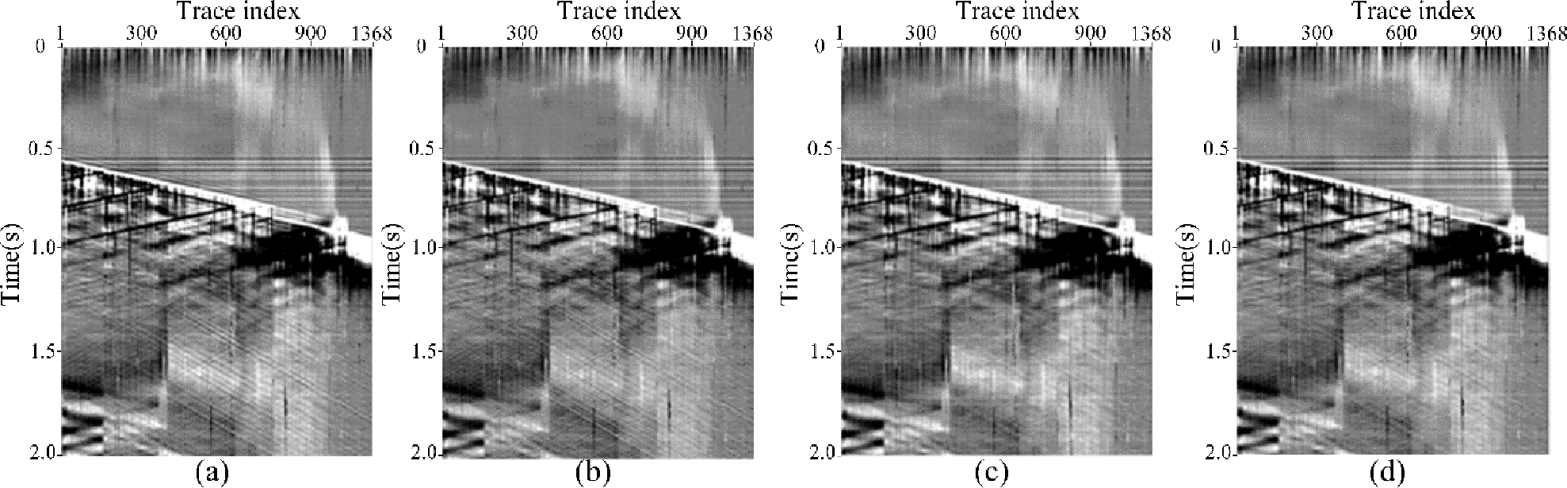
Removed noise by different methods. (a)-(d) Removed noise by bandpass filtering, DnCNN, U-Net and HMR-Net.

To analyze the preservation ability of effective signals, the removed noise is extracted, as shown in Fig 10. Figs 10(a)–10(d) respectively illustrate the residual noise of bandpass filtering, DnCNN, U-Net, and HMR-Net. The bandpass filtering retains numerous valuable signal remnants within the removed noise and exhibits noticeable distortion. Similarly, DnCNN’s noise removal leads to significant signal leakage, indicating excessive noise retention in its denoised results. Meanwhile, both U-Net and HMR-Net (see Figs 10(c) and 10(d)) exhibit more complete noise denoising ability. In comparison, HMR-Net leaves fewer signal residuals in the noise-removed results, further highlighting its denoising and signal preservation capabilities. Moreover, we also calculate the structural similarity index(SSIM) [27] between the denoising results and filtered noise, as shown in Fig 9 and 10. As we know, the value of SSIM may turn to be significant when obvious signal or noise leakage occured. The corresponding results is shown that that for bandpass filtering is 0.3311, indicating limited signal preservation ability. Meanwhile, the SSIMs of DnCNN, U-Net and HMR-Net are 0.1417, 0.1019 and 0.0834, respectively. These results demonstrate the effectiveness of CNN-based frameworks in intense DAS background noise attenuation. Among them, HMR-Net has the smallest value. Therefore, we can infer that HMR-Net can effectively eliminate the intense noise without obvious signal leakage.

## 6. Discussion

### 6.1. Comparison with DeepSeg

**Fig 11:**
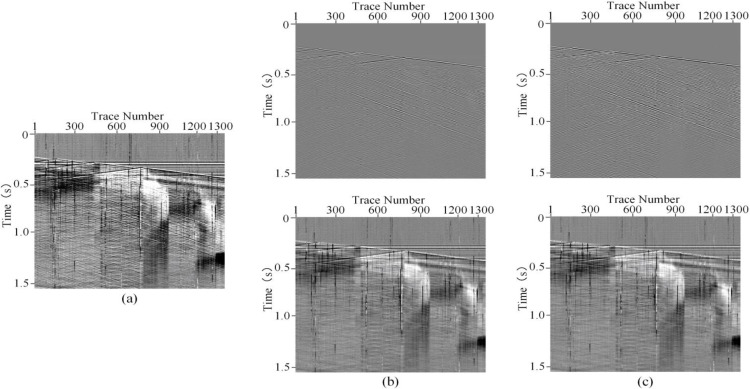
Denoising results of a field DAS data acquired from another survey areas. (a) Field DAS data. (b) and (c) Results obtained by DeepSeg and HMR-Net.

**Fig 12:**
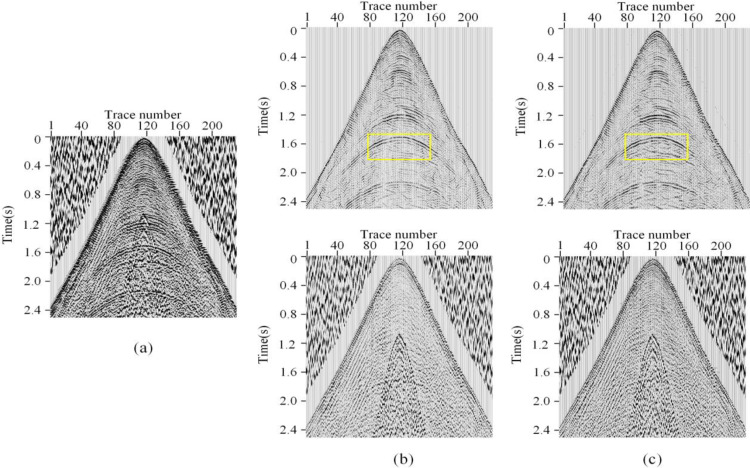
Denoising results of a geophone-acquired seismic data. (a) Field seismic data. (b) and (c) Results obtained by DeepSeg and HMR-Net.

To further investigate the generalization ability of HMR-Net, a real DAS data acquired from another survey areas (Fig. 11(a)) is processed. Notably, the DAS data shows a distinct fasion with the real record directed in Fig. 9. On this basis, DeepSeg [26], a recently proposed framework, is used as the comparing method. The corresponding denoising results are shown in Fig. 11. By observing the figures, we can find that our framework can provide better denoising performance than DeepSeg in the recovery of detailed signals. Moreover, seismic data collected by conventional geophone arrays is also processed. Notably, the effective signals in conventional seismic data have different fundamental frequencies, and the noise also shows different patterns. To achieve better denoising performance, we rebuild the training dataset, composed of synthetic record and field noise data. The corresponding results are shown in Fig. 12. It is shown our proposed framework indicates better ability than DeepSeg. Therefore, we can get the point that HMR-Net has generalization ability and can be applied to seismic data having different properties.

### 6.2. Computational burden analysis

Specifically, the number of convolutional layers of HMR-Net is determined by experiments, which aims to balance the denoising performance and computational burden. The SNR increments are shown in the following Table 4. It is shown that the SNR is not significantly improved with the increasing of convolutional layers in USM and MREM. Therefore, the number of convolutional layers for USM and MREM are selected as 10 and 8, respectively. Hence, the total number of convolutional layers of HMR-Net is 28. Moreover, the training and testing process are conducted using the Matconvnet package on an Intel(R) Core(TM) i5-9400f CPU and an NVIDIA Geforce RTX 2060 Super GPU. The training cost and improved SNR results are shown in Table 5. Notably, the advantages of CNN-frameworks are based on the training cost, which for HMR-Net is almost 4 hours. Another issue need pay attention is that the processing time for these frameworks is relative fast. Such as the processing time for HMR-Net is only 0.177 seconds. Therefore, we can get the point that HMR-Net can provide the impressive denoising capability (SNR increments over 18dB) with an acceptable computational cost.

**Table 4:** Determination of network depth for USM and MREM.

Number of convolutional layers	6	8	10	12	14
USM	13.06	14.67	15.26	15.31	15.40
MREM	13.13	14.56	14.70	14.81	14.90

**Table 5:** Processing efficiency analysis.

Contents	BP Filter	DnCNN	U-Net	HMR-Net
Training time (hours)	0	2.31	3.39	3.98
Processing time (seconds)	0.034	0.151	0.172	0.177
Improved SNR (dB)	0	10.47	16.61	18.14

## 7. Conclusion

In recent years, DAS technology has found widespread application in seismic exploration. However, the interference of random and coherent noise often disrupts the continuity of valuable signals. This paper introduces the HMR-Net denoising network, composed of a U-shaped module and a Multi-Resolution (MR) feature extraction module. The U-shaped module effectively extracts rich shallow and deep features, while the MR module captures features at multiple resolutions, enabling more efficient extraction of underlying data characteristics. Additionally, the up-sampling and down-sampling modules aim to mitigate data loss caused by the sampling operations. Furthermore, to ensure effective training, the training dataset is a combination of synthetic clean data and real seismic noise, incorporating various field noise. This targeted optimization ensures the network’s generalizability for seismic denoising tasks. Through testing on synthetic and real seismic data and comparison with common denoising methods, the proposed approach excels in both effective signal recovery and noise suppression. It is shown that HMR-Net can significantly improve the performance when confronted with DAS data having extractly low SNR. Taking the denoising results of synthetic data with -10dB as an instance. Notably, HMR-Net can significantly attenuate the DAS background noise, offering an SNR increment near 18dB for the synthetic dataset used in this study. Compared with other competing methods, HMR-Net can provide the best denoising performance. In summary, this method effectively preserves valuable signals while mitigating the influence of diverse random and coherent noises in seismic denoising applications.
